# Increased expression of *RXRα *in dementia: an early harbinger for the cholesterol dyshomeostasis?

**DOI:** 10.1186/1750-1326-5-36

**Published:** 2010-09-15

**Authors:** Afia Akram, James Schmeidler, Pavel Katsel, Patrick R Hof, Vahram Haroutunian

**Affiliations:** 1 Department of Neuroscience Mount Sinai School of Medicine, New York, NY 10029, USA; 2 Department of Psychiatry, Mount Sinai School of Medicine, New York, NY 10029, USA; 3 J.J. Peters Veterans Affairs Medical Center, Bronx, NY 10468, USA

## Abstract

**Background:**

Cholesterol content of cerebral membranes is tightly regulated by elaborate mechanisms that balance the level of cholesterol synthesis, uptake and efflux. Among the conventional regulatory elements, a recent research focus has been nuclear receptors, a superfamily of ligand-activated transcription factors providing an indispensable regulatory framework in controlling cholesterol metabolism pathway genes. The mechanism of transcriptional regulation by nuclear receptors such as LXRs involves formation of heterodimers with RXRs. LXR/RXR functions as a sensor of cellular cholesterol concentration and mediates cholesterol efflux by inducing the transcription of key cholesterol shuffling vehicles namely, ATP-binding cassette transporter A1 (ABCA1) and ApoE.

**Results:**

In the absence of quantitative data from humans, the relevance of expression of nuclear receptors and their involvement in cerebral cholesterol homeostasis has remained elusive. In this work, new evidence is provided from direct analysis of human postmortem brain gene and protein expression suggesting that RXRα, a key regulator of cholesterol metabolism is differentially expressed in individuals with dementia. Importantly, RXRα expression showed strong association with ABCA1 and ApoE gene expression, particularly in AD vulnerable regions.

**Conclusions:**

These findings suggest that LXR/RXR-induced upregulation of ABCA1 and ApoE levels may be the molecular determinants of cholesterol dyshomeostasis and of the accompanying dementia observed in AD.

## Introduction

Differential control of gene expression is an important means by which cells respond to physiological and environmental stimuli. Nuclear receptors comprise a superfamily of ligand regulated, DNA-binding transcription factors that can both activate and repress gene expression [1]. The liver X receptors (LXRs) are type II nuclear receptors, initially identified as orphan nuclear receptors, because their natural ligands were not known [2,3]. LXRs have been deorphanized or adopted following the discovery of oxysterols (hydroxylated derivatives of cholesterol) as their endogenous ligands [3,4]. Two LXR isoforms are known, namely LXRα and LXRβ with distinct tissue distributions [5]. LXRα expression is relatively restricted to tissues involved in lipid metabolism, such as liver and intestine [6,7], whereas LXRβ is ubiquitously expressed. Both LXR isoforms are expressed in the brain [5]. LXRβ expression, in particular is 2-5 fold higher in the brain than in liver [8].

**Figure 1:**
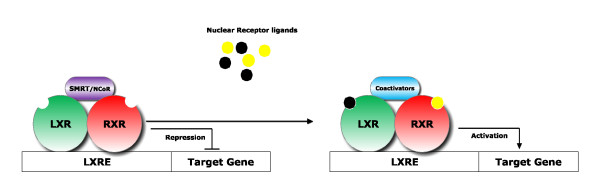
**Schematic diagram of LXR/RXR activation mechanism (adapted from the references cited above)**. In the absence of a ligand, LXR/RXR heterodimers are bound to the LXREs in the promoter region of target genes and in complex with corepressors (SMRT or NCoR). Ligand binding (e.g., oxysterols) induces a dissociation of corepressors and recruitment of coactivators and the target gene expression is induced.

The mechanisms of transcriptional regulation by LXRs involve formation of heterodimers with retinoid X receptor (RXR). RXR is another deorphanized nuclear receptor, activated by 9-cis-retinoic acid (a vitamin A derivative) [9]. Upon heterodimerization, LXR/RXR bind to specific DNA sequences called LXR-responsive elements (LXREs) in the target genes [10]. In the absence of a ligand, LXRs bound to cognate LXREs, are in complex with corepressors such as silencing mediator of retinoic acid and thyroid hormone receptor (SMRT) [11] and nuclear receptor corepressor (N-CoR) [12], consequently, the transcription of target genes is repressed. Receptor ligation induces conformational changes in the ligand binding domain which mechanistically facilitates the release of corepressors and the recruitment of coactivators and histone acetyltransferase (HAT), the enzyme that acetylates lysine amino acids on histone proteins by transferring an acetyl group from acetyl CoA and is generally associated with transcriptional activation [13,14] (Figure 1). Interestingly, LXR/RXR receptors exhibit the "phantom ligand effect," the ability of ligand-induced allosteric signal transmission by nuclear receptors to activate the unliganded heterodimeric partners [15]. These heterodimers are also referred to as permissive because the complex can be activated by ligands of either partner. These unique features allow multiple ligand-mediated pathways to be integrated into a transcriptional response.

Studies over the last decade have established LXRs as master regulators of lipid metabolism. LXR mediates activation of target genes such as sterol responsive element binding protein 1c (SREBP1c), a master transcription factor that controls the entire fatty acid biosynthetic pathway [16], lipid transporters including members of the superfamily of ATP-binding cassette (ABC) transporters such as ABCA1 [17-19], apolipoproteins (ApoE, ApoD) [20,21] and lipoprotein modifying enzymes (cholesteryl ester transfer protein (CETP) and phospholipid transfer protein (PLTP) [22,23]. In addition, primary astrocyte cultures treated with synthetic LXR ligands exhibit increased cholesterol efflux and elevated expression of LXR target genes including ABCA1 and ApoE [8,24,25]. LXRα/β knockout mice show a variety of CNS defects including lipid accumulation, astrocyte proliferation and disorganized myelin sheaths [26,27].

The expression of RXRs has been observed by immunohistochemistry and *in situ *hybridization in mouse brain [28-30]. There are three isoforms of RXRs: RXRα, RXRβ, RXRγ. RXRα and β are most prevalent in the neocortex and hippocampus while RXRγ expression is restricted to the neocortex [31].

Analysis of data from a large-scale microarray study of postmortem brain specimens obtained from multiple brain regions of elderly patients with varying severity of dementia [32] indicated significant changes in RXR gene expression [33,34]. Observed changes in gene expression were isoform-specific with more robust Alzheimer's disease (AD)-associated changes observed in RXRα levels. In particular, the dysregulated expression was most obvious in AD vulnerable regions such as inferior temporal gyrus (area 20) and superior temporal gyrus (area 22) and in the hippocampus, but not in primary visual cortex (area 17) which is relatively spared from age related or AD-associated neurodegeneration [35-40]. In addition, changes in LXR/RXR target genes, ABCA1 and ApoE in AD brains as a function of the increasing severity of dementia and neurofibrillary pathology were also observed [33,34,41]. The significance of these results is twofold. First, expression levels of nuclear receptors and their target genes have not been previously reported in a single cohort of clinically, neuropsychologically and neuropathologically well-characterized AD and control individuals with minimal mRNA variability, known medical history and absence of protracted agonal state. Second, ABCA1 and ApoE are not only LXR/RXR target genes but also the major determinants of net cholesterol flux. These novel findings provided an impetus to study *LXR/RXR *gene and protein expression using more quantitative technique. To this end, we analyzed *LXRβ *and *RXRα *mRNA expression in the hippocampus and inferior temporal gyrus (area 20) of a large series of cases at different stages of dementia and AD associated neuropathology by quantitative PCR. Because mRNA and protein levels can diverge significantly through post-transcriptional regulation, Western blotting was used to quantify protein levels in the hippocampus of a subset of the postmortem brain specimens used in PCR analyses.

## Results

### qPCR analysis of *RXRα *expression in area 20

**Figure 2:**
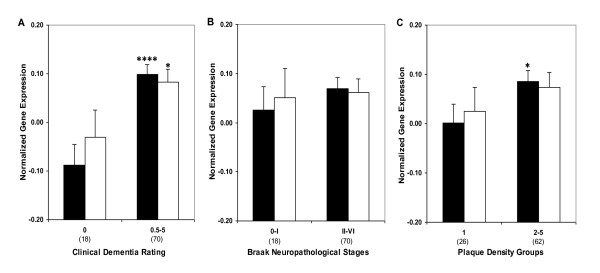
***RXRα *(Black bars) and *LXRβ *mRNA (white bars) expression in individuals with and without dementia or AD-associated neuropathology in area 20**. Mean values ± SEM are shown. *, *p *< 0.05; ****, *p *< 0.0001. Number within the parentheses indicates the individuals within each group.

**Figure 3:**
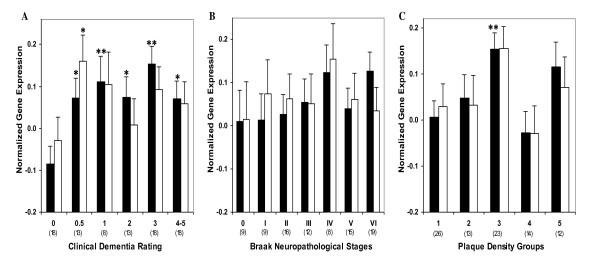
**Normalized *RXRα *(Black bars) and *LXRβ *mRNA (white bars) expression in area 20 plotted against CDR scores, Braak neuropathological stages and NP density**. ANCOVA was used to compare gene expression in individuals with varying degree of dementia (CDR 0.5-5) and AD associated neuropathology (Braak stage I-VI, NP density 2-5) relative to the control group. Mean values ± SEM are shown. *, *p *< 0.05; **, *p *< 0.01. Number within the parentheses indicates the individuals within each disease severity group.

In order to perform post-assay analyses based on a clinical index of disease severity, the subjects were classified with respect to the clinical dementia rating (CDR) score at the time of death (Table 1). Comparison of individuals with and without dementia {i.e., CDR 0 vs. CDR 0.5-5) showed higher levels of *RXRα *gene expression in individuals with dementia (F_1,84 _= 15.48, *p *< 0.0005; Figure 2). Comparisons of individuals with and without AD-associated neuropathology also showed more *RXRα *gene expression as a function of increasing neuritic plaque (NP) density (F_1,84 _= 3.36, *p *= 0.035) but not Braak neuropathological stages (F_1,84 _= 1.42, *p *= 0.237; Figure 2). These results suggest that RXRα expression is dysregulated in the earliest quantified stage of dementia and AD-associated neuropathology. The partial correlations of *RXRα *mRNA expression demonstrated significant associations with CDR (r = 0.301, df = 84, *p *= 0.005). However, the linear association of *RXRα *mRNA levels with either Braak neuropathological stages (r = 0.193, df = 84, *p *= 0.076) or NP density (r = 0.132, df = 84, *p *= 0.225) was not significant, indicating that *RXRα *levels are elevated at the earliest stages of dementia and remain near maximally elevated throughout the course of dementia. In ANCOVAs controlling for age and RNA integrity number (RIN), CDR (F_5,80 _= 3.62, *p *= 0.005) and NP density (F_4,81 _= 3.55, *p *= 0.010) showed significant associations with *RXRα *mRNA expression. Whereas, the association of Braak neuropathological stages with *RXRα *gene expression was not significant (F_6,79 _= 0.85, *p *= 0.536). Figure 3 presents the estimated means and SEM from the ANCOVAs, adjusting for the covariates.

**Table 1:** Group classifications for gene expression analyses.

Gene Expression Analysis			
		**Number of individuals**	
**CDR Groups**	**Dementia Severity**	**Hippocampus**	**Area 20**

0	No dementia	18	18
0.5	Questionable dementia	13	13
1	Mild dementia	9	8
2	Moderate dementia	9	13
3	Severe dementia	12	18
4-5	Very severe/terminal dementia	12	18

**Braak Groups**	**Braak stages**		

0	None	7	9
I	Mild transentorhinal	8	9
II	Severe transentorhinal	15	16
III	Limbic	10	12
IV	Limbic/Hippocampal CA1	8	8
V	Isocortical	12	15
VI	Isocortical/Primary sensory areas	13	19

**NP Density Groups**	**Plaques (number/mm**^**2**^**)**		

0	0	24	26
1	1-5	10	13
2	6-10	20	23
3	11-20	14*	14
4	21 and more	5*	12

### qPCR analysis of *LXRβ *expression in area 20

Gene expression analysis in controls and individuals with dementia (CDR ≥ 0.5) or AD-associated neuropathology showed higher levels of *LXRβ *gene expression (F_1,84 _= 3.14, *p *= 0.040) only in individuals with varying dementia severity (Figure 2). The partial correlation analysis showed that after controlling for age and PMI, *LXRβ *mRNA expression was not significantly associated with CDR (r = 0.045, df = 84, *p *= 0.684), Braak neuropathological stages (r = 0.007, df = 84, *p *= 0.951) or NP density criteria (r = 0.032, df = 84, *p *= 0.771). There was a significant increase in *LXRβ *gene expression with the earliest signs of dementia (CDR 0.5), however, in ANCOVAs the association of CDR (F_5,80 _= 1.30, *p *= 0.274) with *LXRβ *gene expression was not significant. Similarly, the association of Braak neuropathological score (F_6,79 _= 0.30, *p *= 0.938) or NP density (F_4,81 _= 1.83, *p *= 0.132) with *LXRβ *gene expression was not significant. Figure 3 presents the estimated means and SEM from the ANCOVAs, adjusting for the covariates.

### qPCR analysis of *RXRα *expression in hippocampus

**Figure 4:**
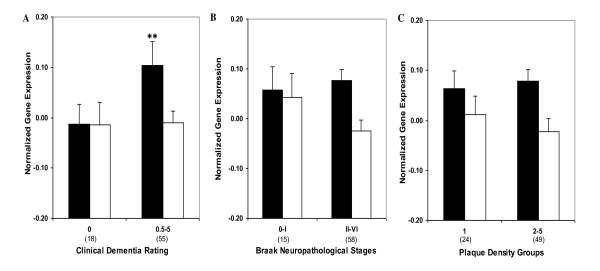
***RXRα *(Black bars) and *LXRβ *mRNA (white bars) expression in individuals with and without dementia or AD-associated neuropathology in hippocampus**. Mean values ± SEM are shown. **, *p *< 0.01. Number within the parentheses indicates the individuals within each group.

**Figure 5:**
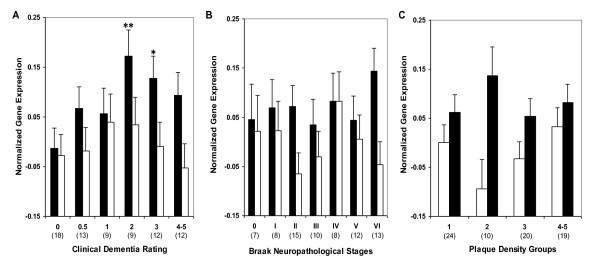
**Normalized *RXRα *(Black bars) and *LXRβ *mRNA (white bars) expression in hippocampus plotted against CDR scores, Braak neuropathological stages and NP density groups**. ANCOVA was used to compare gene expression in individuals with varying degree of dementia (CDR 0.5-5) and AD-associated neuropathology (Braak stage I-VI, NP density 2-5) relative to the control group. Mean values ± SEM are shown. *, *p *< 0.05; **, *p *< 0.01. Number within the parentheses indicates the individuals within control groups and each of the disease severity group.

Comparisons of controls and cases presenting with varying degree of dementia or AD-associated neuropathology showed higher levels of *RXRα *gene expression associated with increases in CDR (F_1,69 _= 6.14, *p *= 0.008) but not with Braak neuropathological staging (F_1,69 _= 0.15, *p *= 0.703) or NP density (F_1,69 _= 0.52, *p *= 0.474; Figure 4). *RXRα *mRNA expression demonstrated significant linear associations with CDR (r = 0.251, df = 69, *p *= 0.035) but not with Braak neuropathological stages (r = 0.127, df = 69, *p *= 0.290) or NP density (r = 0.021, df = 69, *p *= 0.861). In ANCOVAs controlling for age and pH, when CDR (F_5,65 _= 1.83, *p *= 0.119), Braak neuropathological stages (F_6,64 _= 0.59, *p *= 0.740) and NP density (F_3,67 _= 0.53, *p *= 0.667) were treated as categories, the associations were not significant. Figure 5 presents the estimated means and SEM from the ANCOVAs, adjusting for the covariates. Overall, changes in *RXRα *gene expression aligned significantly with clinical measure of the disease compared to the neuropathological parameters.

### qPCR analysis of *LXRβ *expression in the hippocampus

Gene expression analysis in controls and cases with dementia or AD-associated neuropathology did not reveal significant difference in *LXRβ *gene expression (Figure 4). Similarly, the partial correlations of *LXRβ *mRNA expression did not show association with CDR (r = 0.025, df = 70, *p *= 0.835), Braak neuropathological stages (r = 0.029, df = 70, *p *= 0.809) or NP density (r = 0.116, df = 70, *p *= 0.334). In ANCOVAs controlling for age, when CDR (F_5,66 _= 0.48 *p *= 0.794), Braak neuropathological stages (F_6,65 _= 0.94, *p *= 0.472) and NP density (F_3,68 _= 1.27, *p *= 0.290) were analyzed as categories, the lack of associations were similarly evident. Figure 5 presents the estimated means and SEM from the ANCOVAs, adjusting for the covariates.

### RXRα protein expression

**Figure 6:**
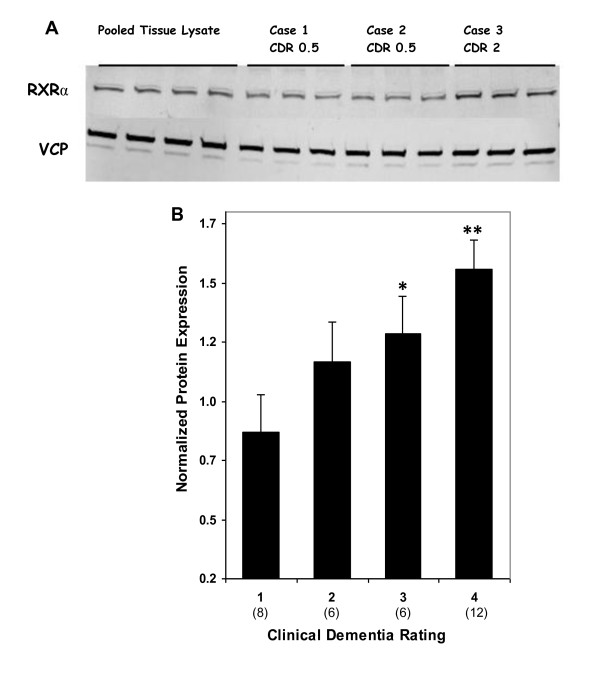
**Western blot analysis of RXRα in the hippocampus of cognitively intact controls and subjects with varying severity of dementia**. A, Representative immunoblots of RXRα protein expression are shown. Total tissue homogenates were separated by reducing SDS-PAGE and probed with rabbit anti-RXRα and mouse anti-VCP antibodies. Tissue lysate from each subjects were loaded in triplicate and pooled tissue lysate (first 4 lanes) were run in quadruplicates. B, Protein quantification was done by assessing the ratio of RXRα and VCP signal. Mean values ± SEM are shown. *, *p *< 0.05; **, *p *< 0.01. Number within the parentheses indicates the individuals within each group.

Western blot analysis revealed robust RXRα protein expression in tissue homogenates from the hippocampus (Figure 6A). RXRα protein expression was highly correlated (r = 0.616, df = 29, *p *< 0.0005) with mRNA levels of *RXRα*. These findings suggest coordinated transcriptional and translational modulation of RXRα during the course of AD. Partial correlation analysis of RXRα protein expression showed strong associations with CDR (r = 0.525, df = 29, *p *= 0.002) but not with Braak scores (r = 0.266, df = 29, *p *= 0.147) or NP density (r = 0.077, df = 29, *p *= 0.681). Even after controlling for Braak score and NP density, RXRα protein expression was significantly correlated with CDR (r = 0.500, df = 27, *p *= 0.006). As was the case for gene expression, in ANCOVAs for protein expression, there was significant association with CDR (F_3,27 _= 3.65, *p *= 0.025; Figure 6B), but not with Braak scores (F_3,27 _= 0.874, *p *= 0.467) or NP density (F_1,29 _= 0.172, *p *= 0.681). Significantly more RXRα protein expression was observed in cases with dementia (CDR ≥ 0.5) relative to controls (CDR = 0) (F_1,29 _= 8.27, *p *= 0.007).

### Association with *ABCA1, ApoE, and LRP *gene expression

**Figure 7:**
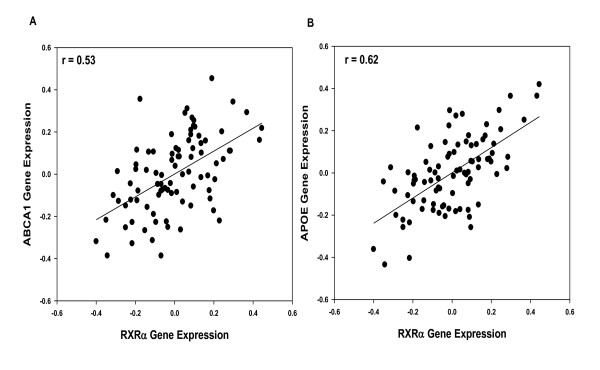
**Association between *RXRα *and target gene expression in area 20**. A. *RXRα *gene expression is strongly associated with *ABCA1 *gene expression (r = 0.531, df = 83, *p *< 0.0001). B. *RXRα *gene expression is strongly associated with *ApoE *gene expression (r = 0.622, df = 84, *p *< 0.0001).

**Figure 8:**
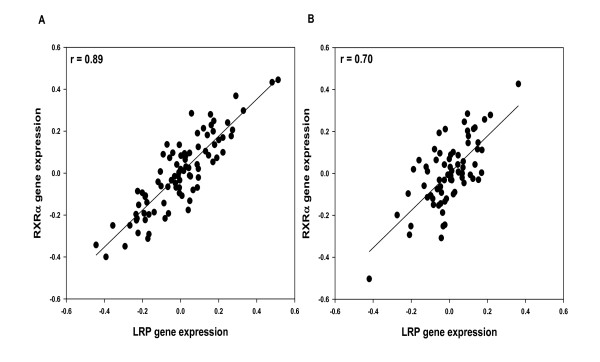
**Association between *RXRα *and *LRP *gene expression**. A. *RXRα *gene expression is strongly associated with *LRP *gene expression in area 20 (r = 0.89, df = 84, *p *< 0.0001). B. *RXRα *gene expression is strongly associated with *LRP *gene expression in hippocampus (r = 0.70 df = 69, *p *< 0.0001).

The association of ABCA1, ApoE and LRP, the major determinants of net cholesterol flux, with *RXRα *mRNA expression was determined using partial correlation analysis. *RXRα *mRNA expression indicated strong association with mRNA levels of *ABCA1 *(r = 0.531, df = 83, *p *< 0.0001; Figure 7A), *ApoE *(r = 0.622, df = 84, *p *< 0.0001; Figure 7B) and LRP in area 20 (r = 0.888, df = 84, *p *< 0.0001; Figure 8A). *RXRα *gene expression in the hippocampus also showed strong associations with ABCA1 (r = 0.436, df = 69 *p *< 0.0005), ApoE (r = 0.446, df = 69 *p *< 0.0001) and LRP gene expression (r = 0.697, df = 69 *p *< 0.0001; Figure 8B).

## Discussion

This study explored the expression profile of the master regulators of lipid metabolism in two of the most vulnerable regions of the AD brain. The primary finding was that RXRα levels increased at the very earliest stage of dementia and remained elevated, in general, throughout the course of the disease. In addition, these elevations in gene and protein expression were more strongly associated with the development of AD-associated dementia than with the measures of mature neuropathologic lesions of AD. In order to identify earliest transcriptional changes during the course of AD progression, study individuals were grouped based on dementia severity (CDR score) at the time of death, progression of NFT pathology and the severity of NP pathology. This strategy allowed us to correlate expression of nuclear receptors to cognitive and pathological indices of AD, with an emphasis on individuals presenting earliest signs of AD. These approaches revealed a previously unrecognized transcriptional dysregulation of *RXRα *in AD. Specifically, alteration in gene expression in both regions was strongly correlated with cognitive impairment. Additionally, we observed highly coordinated upregulation of RXRα protein in AD hippocampus. Along with the identification of altered *RXRα *expression, this study highlights closely correlated expression of RXRα with the downstream target genes that have been previously implicated in AD pathogenesis including *ApoE *and *ABCA1*. One parsimonious interpretation of these findings is that changes in the expression of RXR become evident before the advent of the neuropathological hallmarks of AD and raise the possibility that the upregulation of RXR may be responsible for the changes in subsequent progressive cholesterol dyshomeostasis and AD neuropathology. However, because of the postmortem nature of the current study, the results of this study do not address directly whether the elevated levels of LXR/RXR in dementia are causal, secondary or bystander. It is also important to note that although we interpret transcriptional changes as a function of the severity of dementia or neuropathology to represent disease progression, the cross-sectional nature of this postmortem study, like all postmortem studies, permits only the inference of progression rather than its direct measurement. In addition, the reported results are based on assays of brain tissue homogenates, and therefore cannot inform on the cellular localization of the dysregulated gene expression or the laminar identity of the affected cells.

Although the pathogenesis of AD is not fully understood much direct and circumstantial evidence suggests that many of the genes and pathways involved in cholesterol and/or lipoprotein metabolism in brain are also intimately involved in the pathogenesis of AD. Most notable are ApoE, the principal cholesterol carrier protein in the CNS, and ABCA1, a protein that modulates the efflux of cellular cholesterol and phospholipids to lipid-deficient apolipoprotein acceptors such as ApoE [42-44]. Interestingly, we have observed significant increase in *ABCA1 *expression in the same cohort and tissues specimens as those described here [33,34,41]. Although, in the hippocampus, significant increase in RXRα mRNA level was observed in individuals with moderate to severe dementia, significant increase in RXRα protein expression was evident in individuals at an earlier stage of cognitive decline. Consequently, even small changes in RXRα mRNA expression potentially result in relatively large changes in RXRa protein. Because *RXRα *expression is strongly correlated with transcriptional changes in both *ABCA1 *and *ApoE*, it is conceivable that the variance in RXRα, which is not enough for strong association with CDR to be detected, could nevertheless induce expression of the target genes to the extent that significant correlations with earliest cognitive and neuropathological markers are obvious. These results are particularly intriguing in light of the fact that both *ABCA1 *and *ApoE *are under transcriptional regulation of the RXR/LXR signaling pathway. Together, these findings suggest that RXR/LXR-mediated alterations in *ApoE *and *ABCA1 *levels can modulate cholesterol metabolism and consequently the risk for AD.

In AD, cholesterol dyshomeostasis has been associated with the processing of amyloid precursor protein (APP) to generate neurotoxic Aβ [45]. Given the critical role played by LXR/RXR in the regulation of cellular cholesterol homeostasis, and their expression in multiple brain types (albeit with significant cell type and brain region expression differences), numerous studies have focused on the ability of LXR/RXRs to regulate amyloidogenic processing of APP. However, contradictory results have been reported on Aβ metabolism following treatment of cultured cells with LXR agonists [24,46,47]. The reason for the discrepancies may be related to differences between the expression of APP under physiologic conditions vs. its expression in mutant cells, and the extent to which membrane cholesterol transport was modified under the different *in vitro *conditions. *In vivo *studies using a synthetic LXR agonist, TO-901317, were more definitive in suggesting decreased Aβ deposition in the brains of APP transgenic mice [48,49]. Interestingly, TO-901317 has been shown to inhibit γ-secretase independent of LXR/RXR activation [50]. In addition, TO-901317 is also a farnesoid X receptor and pregnane X receptor agonist [51,52]. This raises concerns about the specificity of TO-901317 and the interpretation of studies using TO-901317 mediated LXR/RXR activation. Finally, LXR/RXR-mediated cholesterol homeostasis is differentially regulated in mice and humans. These differences are accentuated in studies attributing beneficial outcomes to LXR/RXR-mediated transcriptional regulation. In mice but not in primates, hepatic cholesterol 7α-hydroxylase is upregulated by LXR [4,53,54]. Mice do not express CETP. Direct induction of CETP in human cell lines and two CETP-containing animal models, Syrian hamsters and long-tailed macaque monkeys, is accompanied by a significant increase in LDL cholesterol levels that was not previously observed in mice [23,55,56]. These findings emphasize the need to exercise caution when extrapolating results from animal studies to humans, especially when significant species-specific differences have been identified in the underlying biological processes. It is noteworthy that in the current systematic analysis, LXR/RXR expression levels were not associated with elevations in NP counts. It may be plausible that change in RXRα expression may influence soluble toxic forms or fractions of Aβ, yet it does not correlate with NP density because RXRα has a monotonic pattern of expression, whereas NPs continue to increase in density.

LXRs are activated by endogenous oxysterols, the most potent of which include 24, 25-epoxycholesterol, 24-OH, and 27-hydroxycholesterol. The role of 24-OH (also known as cerbrosterol) is particularly intriguing because it is primarily produced in the brain by neuron specific CYP46 [57] and its level is increased in plasma and CSF during early stages of AD [58,59]. 24-OH has been shown to activate LXRs and dramatically elevate ABCA1 expression in both neurons and astrocytes [8,25,60]. Therefore increased expression of ABCA1 and ApoE in incipient AD might be a reflection of 24-OH mediated increased LXR/RXR activation. Paradoxically, 24-OH level is reduced in late stages of AD [61], suggesting that sustained increases in the expression of LXR/RXR target genes in relatively late stages is modulated by other factors.

LXRβ heterodimer with RXRα is the only nuclear receptor complex known to date that can be activated in the absence of a ligand, via a mechanism termed "dimerization-induced activation" [62-64]. In this model of LXR transactivation, the interaction of RXR with LXR can allosterically activate LXR by inducing a conformational change in its ligand-binding domain. The relative expression levels of both receptors are therefore likely to regulate signaling via LXRβ and RXRα in a highly complex fashion. Indeed, in transient transfection studies LXR/RXR activation is only observed upon RXRα cotransfection [3,65,66], which results in a higher number of LXRβ/RXRα heterodimers for which coregulators would have to compete. Because the activated LXRβ/RXRα heterodimer also exhibits dual ligand permissiveness and synergism [62,67], its net transcriptional potential depends on the occurrence of dimerization-induced activation and ligand availability. Consistent with this idea, the overexpression of RXRα reported here could allosterically activate LXRβ and consequently increase target gene expression, in addition to that induced by 24-OH. Recently, LRP has been shown to participate in ABCA1 expression by relieving LXR/RXR repression (via cPLA_2 _activation) [68]. Strong association of LRP gene expression with that of LXR and RXR suggests that LRP modulates not only LXR/RXR activation but also their transcription. Alternatively, LRP may be an LXR/RXR target gene.

## Conclusions

Based on previous studies where a drastic reduction in cholesterol level decreased Aβ production [69-71] and owing to their ability to induce expression of genes mediating cholesterol efflux, LXR/RXR heterodimers have emerged as potential targets for AD therapeutics [72]. However, there are obvious contradicting reports of increased Aβ generation upon lowering brain cholesterol level [73,74]. More importantly, the hippocampus of AD cases presents a moderate, yet significant, reduction in membrane cholesterol [75]. These latter findings are consistent with increased expression of RXRα reported here. Taken together, increased expression of RXRα and concomitant activation of LXR/RXR can modulate ABCA1 and ApoE gene expression. Increased levels of ABCA1 and ApoE may be the molecular determinants of cholesterol dyshomeostasis and accompanying dementia observed in AD.

## Materials and methods

### Study Cohort

The cohort included in this study was part of a larger clinical and neuropsychological investigation of early AD. These individuals were extensively evaluated for their cognitive function. Their cognitive status during the 6 months proximal to death was used to define the absence, presence and extent of dementia at the time of death, as previously described [76-78]. Cases were selected from a pool of over 600 donors with either no discernable neuropathology or only those neuropathological lesions associated with AD alone (e.g., exclusion of cases with vascular lesions, Lewy body inclusions, normal pressure hydrocephalus). Because postmortem intervals (PMI) [79,80] and tissue pH (a proxy measure for agonal state) [81,82] are important issues for consistency and reproducibility of quantitative gene and protein expression studies, brain samples were included from cases who met the following criteria only: postmortem delay of less than 24 hours, brain tissue pH of 6.3 or greater no perimortem coma longer than 6 hrs, no evidence of seizures in the 3 months preceding death. Controls were derived from persons who, on extensive medical record review and/or neuropsychological examination and caregiver interview, showed no evidence of neurological or neuropsychiatric diseases, died of natural causes (myocardial infarction, various non-brain non-hepatic cancers, and congestive heart failure) and had no discernable neuropathology [83]. None of the subjects had a history of licit or illicit drug abuse (tobacco use excepted). All diagnostic and cognitive assessment procedures were approved by the Mount Sinai Medical Center (New York, NY)/J. J. Peters Veterans Administration Medical Center (Bronx, NY) Institutional Review Boards, and postmortem consent for autopsy and research use of tissue was obtained from the next of kin or a legally authorized official.

### Classification of Subjects into Dementia Severity Groups

In order to perform post-assay analyses based on a clinical index of disease and dementia everity, the subjects were classified with respect to the CDR score at the time of death [84-87] (Table 1). CDR is a scale that objectively stages dementia severity from 0-5 with 0 representing no dementia, 0.5 representing questionable dementia or mild cognitive impairment and 1-5 representing gradations of dementia severity from mild to terminal. The assessments, on which these classifications were based, were performed blind to clinical or neuropathological disease diagnosis. Table 2 describes the sample size, sex, age at the time of death, pH and PMI of the study cohort when grouped on the basis of CDR.

**Table 2:** Demographic details of study cohort stratified with respect to CDR (Clinical Dementia Rating) groups.

Characteristics	Area	CDR 0	CDR 0.5	CDR 1	CDR 2	CDR 3	CDR4-5
Total subjects*	Hipp	18	13	9	9	12	12

	Area 20	18	13	8	13	18	18

Gender (men/women)	Hipp	7/11	6/7	3/6	0/9	3/9	3/9

	Area 20	6/12	7/6	3/5	1/12	7/11	8/10

Age (years)	Hipp	75.2 ± 3.5	85.4 ± 2.7	83.4 ± 3.4	87.9 ± 2.0	88.8 ± 1.7	85.0 ± 1.9

	Area 20	77.0 ± 3.9	85.5 ± 2.8	85.6 ± 3.8	87.6 ± 2.0	86.2 ± 8.5	84.2 ± 2.5

Brain pH	Hipp	6.43 ± 0.04	6.43 ± 0.07	6.31 ± 0.1	6.38 ± 0.09	6.34 ± 0.05	6.39 ± 0.07

	Area 20	6.42 ± 0.05	6.42 ± 0.07	6.35 ± 0.11	6.39 ± 0.08	6.43 ± 0.05	6.36 ± 0.05

RNA integrity number (RIN)	Hipp	6.6 ± 0.1	6.2 ± 0.1	6.2 ± 0.2	6.2 ± 0.2	6.1 ± 0.1	6.2 ± 0.1

	Area 20	6.9 ± 0.1	6.9 ± 0.1	6.7 ± 0.2	6.3 ± 0.2	6.9 ± 0.2	6.3 ± 0.1

Postmortem interval (minutes)	Hipp	713 ± 137	393 ± 85	264 ± 39	336 ± 66	276 ± 40	332 ± 80

	Area 20	574 ± 109	381 ± 91	325 ± 52	358 ± 64	244 ± 29	310 ± 73

### Neuropathological Assessment

The neuropathological assessment procedures used have been previously described in detail [76,78]. Neuropathological assessments were performed on the right hemisphere and consisted of microscopic assessment of paraffin embedded blocks from multiple brain regions using hematoxylin and eosin, modified Bielschowski, modified thioflavin S, and anti-amyloid, anti-tau tau when necessary. All neuropathology data regarding the extent and distribution of neuropathologic lesions were collected blind to the subject's dementia status. Specimens for this study were dissected from the frozen, never-thawed, left hemisphere, using previously described procedures [88].

For pathologic staging of AD neurofibrillary tangle density was assessed using the Consortium to Establish a Registry for Alzheimer's Disease (CERAD) [89,90] criteria, NFTs were evaluated using the criteria by Braak and Braak [91] (Table 1). Neuritic plaques (NPs) were identified as the dystrophic neurites arranged radially and forming a discrete spherical lesion about 30 mm in diameter with amyloid cores. NP groups in Table 1 reflect a composite score of NPs counts in 5 cortical regions. The composite measure of cortical NP density was used to reflect better the general level of disease severity and to match more closely to the global assessment of cognitive function measured by the CDR.

### RNA Isolation

Total RNA was isolated from 50 mg of microdissected pulverized frozen brain samples from inferior temporal gyrus and the hippocampus with the guanidinium isothiocyanate method [92] using ToTALLY RNA kits (Ambion, Austin, TX) according to the manufacturer's protocol as described previously [93]. The quality of the isolated total RNA for each case was assessed using a combination of 260 nm/280 nm ratio obtained spectrophotometrically (Beckman Instruments, Fullerton, CA) and by Bioanalyzer 2100 (Agilent Technologies, Palo Alto, CA) before proceeding with cDNA synthesis. Only specimens with an RIN ≥ 5.5 were included in the analyses.

### Reverse Transcriptase Reaction

cDNA synthesis was performed with iScript cDNA Synthesis kit (BioRad Laboratories, Hercules, CA) which uses both random and poly-dT priming for the reverse transcription (RT) reaction. Total RNA (1 μg) was employed for each 20 μl reaction. The resulting cDNA was diluted 25 times for qPCR.

### qPCR

*LXRβ *and *RXRα *mRNA expression was measured by quantitative polymerase chain reaction (qPCR) using an ABI Prism 7700 Sequence Detector (Applied Biosystems, Foster City, CA) and gene-specific fluorogenic TaqMan^® ^probes (Applied Biosystems). Each 20 μl PCR reaction contained 5 μl of the relevant cDNA, 20X TaqMan^® ^assay (used at a final concentration of 0.5X), and 10 μl of TaqMan^® ^Universal PCR Reaction Mix which contains ROX as a passive internal reference (Applied Biosystems). The thermal cycling program consisted of 2 min at 50°C, 10 min at 95°C, followed by 40 cycles of 15 s at 95°C and 1 min at 60°C. The reactions were quantified by selecting the amplification cycle when the PCR product of interest was first detected (threshold cycle, Ct). Tests of primers and probes sensitivity and assay linearity were conducted for all real-time PCR assays by amplification of mRNA in 10-fold serial dilutions of pooled as previously described [94]. Each reaction was performed in triplicate and the average Ct value was used in all analyses.

The relative gene expression level was calculated using the Relative Standard Curve Method (see Guide to Performing Relative Quantitation of Gene Expression Using Real-time Quantitative PCR, Applied Biosystems). Standard curves were generated for target assay and for each endogenous control assay by the association between the Ct values and different quantities (5 serial dilution steps) of a "calibrator" cDNA. The "calibrator" was prepared by mixing small quantities of all experimental samples. Expression values of the target and the control genes were extrapolated from their respective standard curves. Relative expression of target genes was computed as the ratio of the target mRNA levels to the geometric mean of the four endogenous controls: β-glucuronidase (*GUSB*), cyclophilin A (*PP1A*), β2-microglobulin (*β2M*), and ribosomal protein, large, P0 (*RPLP0*) which were picked for their stability using geNorm [95,96]. Samples with Ct values > 33 were considered outside the range of sensitivity of the assay and were not included in the analyses.

### Protein Quantitation

Protein expression studies were carried out to determine whether different levels of *RXRα *gene expression were reflected in the expression level of RXRα protein. Because of the inherently lower reproducibility and higher variability characteristic of Westerns in postmortem tissue relative to qPCR, we restricted RXRα protein analyses to cases with most robust changes in gene expression. Therefore, a subset of the hippocampal samples (N = 30) studied for mRNA expression was analyzed by Western blotting to reflect broad variations in gene expression. As fewer protein analyses were performed than gene analyses, adjacent categories for all three indices of disease severity (see below) for gene expression analyses were combined to achieve sufficiently large sample sizes for comparisons (Table 3).

**Table 3:** Group classifications for protein expression analysis.

Protein Expression Analysis in the hippocampus		
**CDR Groups**	**Dementia Severity**	**Number of individuals**

0	No dementia	8
0.5	Questionable dementia	6
1-2	Mild/moderate dementia	6
3-5	Severe dementia/terminal dementia	10

**Braak Groups**	**Braak stages**	

0-I	None/Mild transentorhinal	8
II	Severe transentorhinal	8
III-1V	Limbic/Hippocampal CA1	7
V-VI	Isocortical/Primary sensory areas	7

**NP Density Groups**	**Plaques (number/mm2)**	

1	0	12
2-5	1 and more	18

### Tissue Lysate Preparation

Total tissue lysates were prepared from frozen hippocampal specimens from sister aliquots of the same brain samples as those used for qPCR analysis as described previously [41]. Total protein concentration of the lysate was determined using a CBQCA Quantitation Kit (Molecular Probes, Eugene, OR) with fluorescence measured on a SpectraMAX Gemini XS spectrofluorometer (Molecular Devices, Sunnyvale, CA).

### Western Blot Analysis

For gel electrophoresis, 10 μg of total protein was mixed with loading buffer and loaded onto pre-cast 10-20% Tris-glycine gels (Bio-Rad Laboratories, Hercules, CA), and run at 150 V for 1 hr. Each gel was loaded with three experimental samples in triplicate and "standard tissue homogenate" (the mix of small aliquots of tissue from all samples), run in quadruplicates. Separated proteins were transferred to polyvinylidene difluoride membranes at 100 V for 1 hour and probed with anti-RXRα antibody (Abcam, Cambridge, MA) diluted 1:5,000 in 3% non-fat dry milk in TBS overnight at 4°C with gentle shaking. To ensure equal protein loading between individual samples, membranes were also incubated with an anti-valosin containing protein (VCP) antibody. VCP, a 97 kDa protein, has been previously validated as reliable internal standard [97]. Following 1 hour incubation with the fluorescently-labeled secondary antibodies, blots were scanned and quantified using the Odyssey IR imaging system (LI-COR Biosciences, Lincoln, NE). RXRα signal was first normalized to the corresponding average signal for the standard tissue homogenate and then for the VCP band from the same sample. The linearity of the dose responses for the antibodies used was established in preliminary experiments.

### Statistical Analyses

We performed a logarithmic transformation of *LXRβ *and *RXRα *gene expression to eliminate heterogeneity, and used the transformed gene expression values for all subsequent statistical analyses. A preliminary analysis assessed linear associations with gender, pH, PMI and RIN to evaluate their use as covariates. In addition, age, the most significant risk factor for dementia and a critical determinant of the extent of AD associated neuropathology, was used as a covariate in all analyses regardless of its association with the dependent variable.

We determined the linear association *LXRβ *and *RXRα *gene expression with CDR, Braak stages and NP density by partial correlation analyses, controlling for potential covariates if preliminary analyses showed significant correlation with the expression level of the gene under analysis. Because the associations of each of these interrelated scales with gene expression is at least partly mediated through the associations with the other two scales, additional partial correlation analyses assessed each scale controlling also for the other two scales.

In order to determine non-linear association of CDR Braak stages, and NP density with *LXRβ *and *RXRα *gene expression, each of these disease severity indices was classified as a categorical variable. ANCOVA was performed for each categorical variable controlling for age and any other potential covariates. Another ANCOVA for each categorical variable controlled also for other two variables as scales, similar to the partial correlation analyses.

Analyses for protein expression were the same as for gene expression. All analyses were performed with SPSS 17.0 (SPSS, Chicago, IL).

## Competing interests

The authors declare that they have no competing interests.

## Authors' contributions

AA carried out the qPCR and Western blot studies and drafted the manuscript. JS performed the statistical analysis. PK, PRH and VH conceived of the study, and participated in its design and coordination and helped to draft the manuscript. All authors read and approved the final manuscript.
